# Targeted nanoencapsulation of tunicamycin reduces toxicity while improving its therapeutic effectiveness in pancreatic cancer cells

**DOI:** 10.1016/j.omton.2025.201047

**Published:** 2025-09-03

**Authors:** Debasmita Dutta, Sunil P. Upadhyay, Archana De, Inamul Haque, Axel H. Breier, Alok De, Daniel J. Mettman, Suman Kambhampati, Mohiuddin Quadir, Francisco Diaz, Sushanta K. Banerjee, Stefan H. Bossmann, Snigdha Banerjee

**Affiliations:** 1 Department of Coatings and Polymeric Materials, North Dakota State University, Fargo, ND 58108, USA; 2 Cancer Research Unit, VA Medical Center, Kansas City, MO 64128, USA; 3 Department of Cancer Biology, University of Kansas Medical Center, Kansas City, KS 66160, USA; 4 Pathology Department, VA Medical Center, Kansas City, MO 64128, USA; 5 Department of Pathology and Laboratory Medicine, University of Kansas Medical Center, Kansas City, KS 66160, USA; 6 Department of Biostatistics and Data Science, University of Kansas Medical Center, Kansas City, KS 66160, USA

**Keywords:** MT: Regular Issue, iRGD, Tunicamycin, nanoparticles, molecular heterogeneity, single-cell phosphoproteomic, CCN1, K-Ras^G12D^, CYR61, KPC model

## Abstract

Pancreatic ductal adenocarcinoma (PDAC) remains one of the leading sources of cancer mortality worldwide. An initial response to chemotherapy, such as gemcitabine (GEM) alone or in combination with other chemotherapies, is often followed by emergent resistance, underscoring the urgent need for targeted therapies. PDAC cells are highly addicted to oncogenic K-RAS mutations for their growth, progression, immunosuppression, and drug resistance, but mutant K-RAS in PDAC is still challenging to target. A N-glycosylation inhibitor, tunicamycin (TM), is a potent killer of PDAC cells. However, the free TM is very toxic in clinical settings. We developed a pH/hypoxia-responsive iRGD-tagged biodegradable nano-encapsulated TM (^NP^TM) that overcomes the limitations of free TM and shows promising results in inhibiting PDAC cell growth via apoptosis. The ^NP^TM has shown significant promise, reducing cellular heterogeneity, drug resistance, *in vitro* desmoplasia, and subcutaneous tumor growth and markedly prolonging the survival in a KPC-xenograft mouse model. The studies suggest that TM targets K-Ras^G12D^-dependent multiple signaling pathways such as EIF4E, STAT3, and STAT5 activities and CCN1 to promote its anticancer efficacy. Together, these studies reveal the potential of simultaneously targeting a K-Ras^G12D^-dependent signal and CCN1 with first-line chemotherapy and provide a rationale for future clinical testing of ^NP^TM for PDAC therapy.

## Graphical abstract

**Figure:**
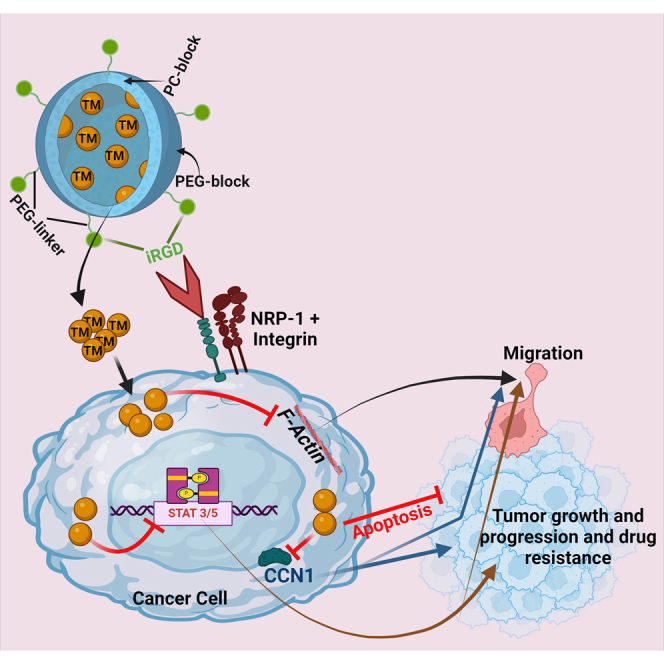

## Teaser

Tunicamycin is a nucleoside antibiotic that inhibits N-glycosylation, effectively targeting cancer cells. However, its clinical use is hampered by off-target toxicity. Our research demonstrates that nanoencapsulation can reduce this toxicity and improve targeted therapy for PDAC. This breakthrough studies will attract global interest in tunicamycin nanoencapsulation for therapies.

## Introduction

Pancreatic ductal adenocarcinoma (PDAC) is the deadliest cancer in the pancreas and constitutes 90% of pancreatic cancers, with a 5-year survival rate of only 10%. Currently, PDAC is the 3^rd^ leading cause of cancer-related death in US populations and is expected to become the 2^nd^ by 2040.^1^^,^^2^ The American Cancer Society revealed that, in 2024, an estimated 66,440 Americans will be diagnosed with pancreatic cancer in the USA, and more than 51,750 will die from the disease (*American Cancer Society: Cancer Facts & Figures 2024 and Hirhberg Foundation for Pancreatic Cancer Research)*. Based on genomic alterations, PDAC can be classified into four groups: stable genomes (<50% structural variants per genome), scattered genomes (50–200 structural variants per genome), locally rear-ranged genomes (>200 structural variants clustered on less than three chromosomes), and unstable genomes (>200 structural variants distributed in the genome).^3^^,^^4^ This genome heterogeneity offers an opportunity for precision oncology for PDAC patients and has, therefore, clinical potential.^3^ However, precision medicine in PDAC, utilizing genome heterogeneity, has not yet been established, underscoring the need for further research and development.^5^

Thus, the standard treatment for PDAC consists of surgical resection (if possible), chemotherapy, immunotherapy, and radiation therapy. Principally, two or several treatment approaches can be combined. Regrettably, the outcome of these treatment options failed to improve overall survival (OS) effectively.^6^ Therefore, the first-line treatments of PDAC are still FOLFIRINOX (combination of 5-fluorouracil, leucovirin, oxaliplatin, and irinotecan) and gemcitabine alone or in combination with nab-paclitaxel (Abraxane), a nanoparticle formulation of paclitaxel.^1^^,^^7^ Studies found that nab-paclitaxel or erlotinib promoted GEM sensitivity, and thus, combination therapy increased the median overall survival (OS) for months, followed by resistance to the drug in most patient populations.^6^^,^^8^ The drug resistance or inadequate delivery in PDAC is due to the complex tumor microenvironment (TME)/tumor ecosystem (TE). Consequently, these therapies are unable to annihilate PDAC.

Nearly 95% of PDAC patients are harboring oncogenic K-RAS mutations (mK-Ras), and K-RAS^G12D^ is the most dominant mutation. It is detected in about 40% or more patients, with shorter survival rates among all PDAC patients.^9^^,^^10^^,^^11^^,^^12^ mK-RAS regulates almost all hallmarks of PDAC, including TME and drug resistance, via multiple downstream signaling pathways.^11^^,^^13^

CCN1, a matricellular glycoprotein and a member of the cellular communication network (CCN) family growth factor, is overexpressed in human PDAC with mK-RAS and found to promote tumor progression via integrin α_V_β_3_ in an orthotopic mouse model.^14^^,^^15^^,^^16^^,^^17^ However, the lack of effective treatments that target CCN1 protein has reached even preclinical studies, highlighting the urgent need for newer drugs. These drugs must have the potency to block CCN1 with less toxicity, a crucial requirement to overcome the limitations of PDAC therapy.

Tunicamycin (TM) is one of the potent nucleoside antibiotics and an inhibitor of aberrant glycosylation in various cancer cells. TM is a potent inhibitor of cancer cell growth and tumor progression.^18^^,^^19^ It disrupts protein maturation by impeding the transfer of UDP-N-acetylglucosamine (GlcNAc) to dolichol phosphate in eukaryotic cells’ endoplasmic reticulum (ER).^19^ Like chemotherapies such as doxorubicin (DOX), 5-fluorouracil, etoposide, and cisplatin, TM induces the unfolded protein response (UPR) by blocking aberrant glycosylation. This leads to stress induction in the ER, which promotes apoptosis via multiple signaling pathways. These include possibly activating the mTORC1-eNOS-RagC pathway*^20^* and the TRAIL-induced pathway.^21^ TM’s potential as a potent antitumor drug in various cancers is significant, but its ability to promote chemosensitivity is a game-changer. However, the non-specificity of TM, causing a life-threatening issue in TM therapy, has led to stagnation in its clinical research. This underscores the urgent need for novel strategies to leverage TM therapeutically in cancer treatment.

The use of nanocarriers/nanoparticles to reduce drug cytotoxicity has attracted significant global attention in the biomedical field.^22^ In this study, we have encapsulated TM (^NP^TM) in pH- and hypoxia-responsive nanoparticles, a method that we have recently established for drug delivery.^23^ These nanocarriers, constructed from amphiphilic-block-copolymer-derived nanoplatforms and conjugated with the iRGD peptide (CRGDKGPDC),^24^ are designed to promote receptor-mediated endocytosis, enhancing selective accumulation in tumor tissues, which leads to improved cellular internalization and *trans*-vascular transport. Finally, encapsulated cargo selectively releases the drugs in acidic pH or hypoxic zones of aggressive tumor tissues, including PDAC.^23^^,^^25^^,^^26^ Mechanistically, under low pH conditions, the tertiary amine groups present within the polymer backbone of the nanoparticles become protonated. This protonation introduces positive charges along the polymer chains, leading to electrostatic repulsion between adjacent polymer segments. As a result, the nanoparticles swell due to increased intermolecular repulsion, creating transient pores and channels through which the encapsulated drug is gradually released. With further acidification, the increased protonation amplifies the repulsive forces, eventually leading to a structural destabilization and catastrophic collapse of the nanoparticle matrix, thereby facilitating a burst release of the remaining drug payload.^23^^,^^27^ These delivery devices are made of bio-compatible, non-toxic, and bio-degradable reagents.^28^^,^^29^^,^^30^ Importantly, our research is the first to demonstrate that this delivery method effectively overcomes the toxicity of TM while enhancing the inhibitory effects on *in vitro* cellular growth and molecular heterogeneity, CCN1 overexpression, and desmoplasia. Unlike unencapsulated TM, ^NP^TM inhibits tumor growth and promotes the survival of tumor-bearing mice with no toxic effects. Therefore, ^NP^TM therapy presents a promising and unique therapeutic approach in K-RAS^G12D^ mutant PDAC, with significant potential to impact cancer treatment.

## Results

### Characterization and detection of cellular uptake of pH/hypoxic-responsive nanoparticles by PDAC cells

**Figure 1:**
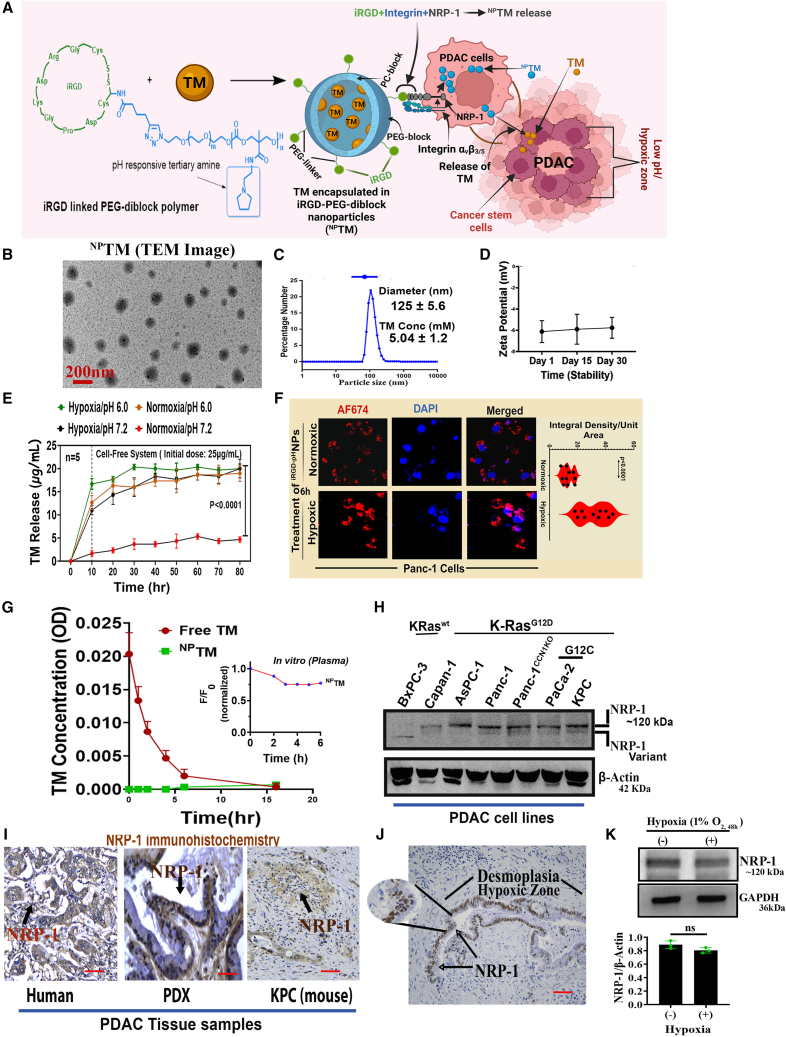
Chemical characterization of ^NP^TM (A) Schematic representation of TM delivery into the PDAC cells using iRGD-tagged pH/hypoxic responsive nanoparticle (^NP^TM). (B and C) PEG-Py nanoparticle size stability detection using TEM micrograph. (D) Stability of the particles measured using Zeta potential. (E) Cumulative TM release profile in cell-free lysis buffer under hypoxic, normoxic, and low pH conditions using controlled CO_2_ incubator. The TM release was measured using UV/VIS spectrophotometer following standardization of the protocols (Figures S2 and 3). *n* = 5. (F) Cells were treated with dye-labeled nanoparticles for 6 h, and cellular distribution was determined using confocal microscopy. The Violin diagram representing the integral density/unit area. (G) *In vitro* stability profile of TM in mouse plasma. Inset shows AF-647-labeled NPs stability. Data represent mean ± SD of *n* = 3. (H & I) NRP-1 levels in PDAC cell lines and tissue samples using WB and IHC, respectively. Original western blot data provided in Figure S5. Scale bars, 50 μm. (J and K) NRP-1 levels in hypoxic and normoxic conditions in tumor tissue (J) and Panc-1 cells (K).

For the last several years, our groups have been actively developing and characterizing targeted pH/hypoxic-responsive block copolymeric nanoparticles (NPs) conjugated with a targeting ligand for drug delivery systems.^23^^,^^31^^,^^32^ Specifically, an amphiphilic block copolymer (Figure S1) conjugated with iRGD (internalizing RGD) peptide (CRGDKGPDC) utilized as a nanocarrier to release the encapsulated drug payload selectively in PDAC within acidic pH or hypoxic zones (Figure 1A), an essential feature of aggressive tumor tissue, including PDAC.^25^ The delivery devices are made of biodegradable and non-toxic nanoparticles.^28^^,^^29^^,^^30^ We want to highlight that the different uptake mechanisms of iRGD-conjugated versus non-conjugated nanoparticles have been thoroughly documented in the literature.^33^ In comparison to non-conjugated nanoparticles, the therapeutic efficacy of iRGD-conjugated nanoparticles significantly enhances drug delivery.^34^^,^^35^ This deficiency may be related to the delivery mechanism. NPs without iRGD typically enter cells through nonspecific pathways, such as fluid-phase or bulk endocytosis, which are significantly less efficient and lack selectivity. In contrast, when NPs are modified with iRGD, they bind to αvβ3/β5 integrins and neuropilin-1 (NRP-1) receptors, enabling a multi-step process for active targeting and improved tissue penetration.^36^ Further, NPs are <125 nm in size (regarding hydrodynamic diameter, DH) and release drugs in the tumors’ hypoxic or low pH zones. Because of these intrinsic biophysical properties, it cannot cross the blood-brain barrier (BBB) or release the TM into the brain, which has a 7.2 pH. Given the extensive published data, we considered iRGD-conjugated pH/hypoxic-responsive nanoparticles for these studies and opted not to repeat those comparative studies in our current work.

Consistent with previous studies,^23^ ^NP^TM showed an average diameter of around 125nm (Figures 1B and 1C), and the particles are long-term stable in aqueous environment (Figure 1D). The functional efficiency test (cell-free release analysis and cellular uptake) showed that TM is efficiently released in hypoxic/low pH (Figure 1E). TM concentrations were measured using a UV/VIS spectrophotometer at a wavelength of 300 nm after standardizing protocols (Figures S2 and 3). We found consistent results when Panc-1 cells were treated with Alexa-Fluor-647-labeled iRGD-tagged pH-responsive NPs. In agreement with previous findings,^37^ cellular uptake increased significantly under hypoxic conditions compared to normoxic environments (Figure 1F).

### *In vitro* plasma stability studies of free and ^NP^TM

To investigate whether nanoencapsulation enhances the stability of TM, we assessed the *in vitro* stability profile of free-TM and ^NP^TM in mouse plasma at various time points. This was followed by measuring TM concentrations using a UV/VIS spectrophotometer at 300 nm (Figures S2 and S3). The *in vitro* plasma stability assay results indicated that no detectable amount of drug was released from the nanoparticles during the first 10 hours of incubation in mouse plasma. In contrast, free-TM was consistently detected in the plasma over the 0- to 16-h period, exhibiting gradual degradation (Figure 1G). This suggests that plasma significantly reduces the stability of free TM. Further, the free TM’s calculated half-life was t_1/2_ = 1.836 h, with a 95% confidence interval of (1.717, 1.955) (Figure S4).

### NRP-1 overexpressed in PDAC cells harbors mutant K-Ras

The NRP-1 expression in tumor cells is necessary for iRGD-mediated targeted drug delivery^38^ (Figure 1A). We sought to determine the expression level of NRP-1 in different PDAC cell lines and tissue samples to validate this drug-targeting concept. We found that the regular form of NRP-1 was highly expressed in all PDAC cell lines harboring mutant K-Ras, except that BxPC3 cells containing wild-type K-Ras expressed a variant form (Figures 1H and S5). In tissue sections collected from human, patient-derived xenograft (PDX), and mouse (KPC) PDAC tumors, we observed a strong NRP-1 immunoreaction (Figure 1I).

Multiple studies have shown that NRP-1 expression was significantly increased in hypoxia-primed cervical and pancreatic cancer cells.^39^^,^^40^ Further, it was also reported that hypoxia-mediated HIF-1alpha-dependent upregulation of NRP-1 is a critical molecular event involved in the vasculogenic mimicry and tumor formation.^41^ However, a contrast effect of hypoxia on NRP-1 expression was also documented,^42^ indicating that the effect of hypoxia on NRP-1 expression could be a context-dependent. Our studies found that the NRP-1 expressions were detected in hypoxic zone of human PDAC tissue samples (Figure 1J) and unaltered in a long exposer of Panc-1 cells in a hypoxic environment (Figure 1K), indicating iRGD-dependent nanoparticles delivery would not be affected.

### IC_50_ is significantly lower in ^NP^TM than free-form TM in PDAC cells

**Figure 2:**
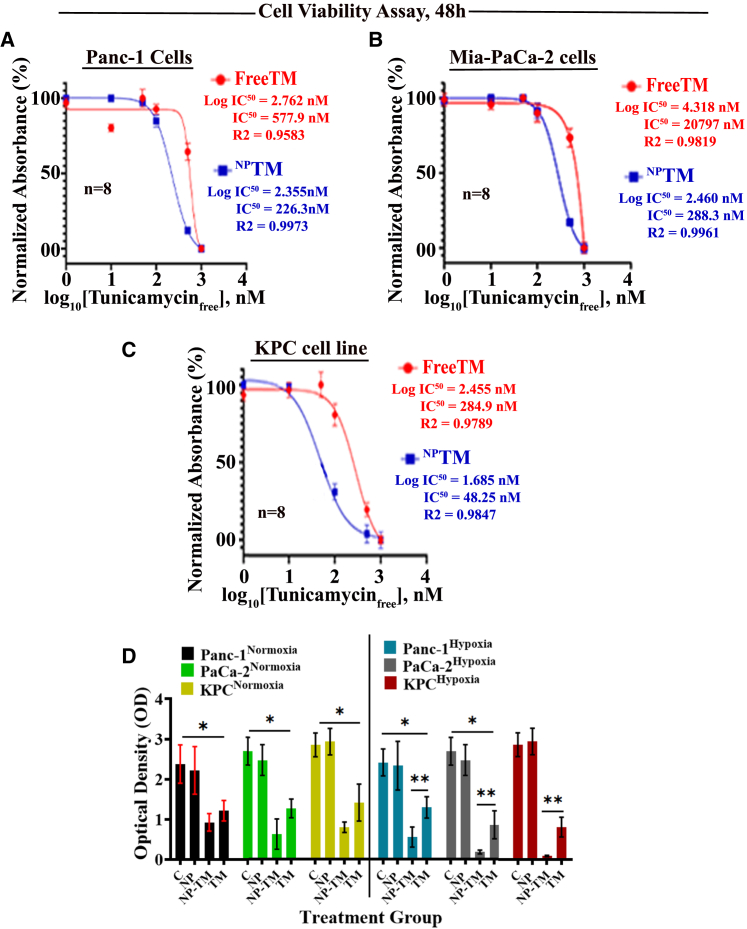
Detection of IC_50_ of free-form TM and ^NP^TM in three PDAC cell lines (A–C) Panc-1, MIA-PaCa-2, and KPC cells were treated with different doses of drugs, stained with crystal violet, and IC_50_ was calculated using GraphPad prism software. (D) Determined the effect of free-TM and ^NP^TM on cell viability under normoxic and hypoxic environments. Cell viability was measured using crystal violet assay. Data represent mean ± SD. ∗*p* < 0.0001 and ∗∗*p* < 0.001; *n* = 5 or 8.

We measured the IC_50_ of free-form TM and ^NP^TM in three different PDAC cell lines: two human PDAC cell lines (Panc-1 and MIA-PaCa-2) and one mouse PDAC cell line (KPC). The results indicate that the IC_50_ of ^NP^TM is significantly lower in all three cell lines compared to unencapsulated free-form TM (Figures 2A–2C). This significant difference in IC_50_ values underscores the potential of ^NP^TM as a potent and safe PDAC inhibitor.^43^

Next, we determined the effect of IC_50_ of ^NP^TM on cell viability under normoxic and hypoxic conditions using a normoxic/hypoxic C0_2_ incubator. Consistent with previous results,^23^ we found no significant difference between untreated and NP-alone-treated cells under normoxic and hypoxic culture media. At the same time, ^NP^TM at a dose of 0.5 μM showed significant inhibition of cell growth than free-TM (Figure 2D). We added an equivalent dose of drug-free NPs as a control that is used in ^NP^TM.

### ^NP^TM-induced inhibition of PDAC growth is dose- and time-dependent and sensitizes the effect of GEM

**Figure 3:**
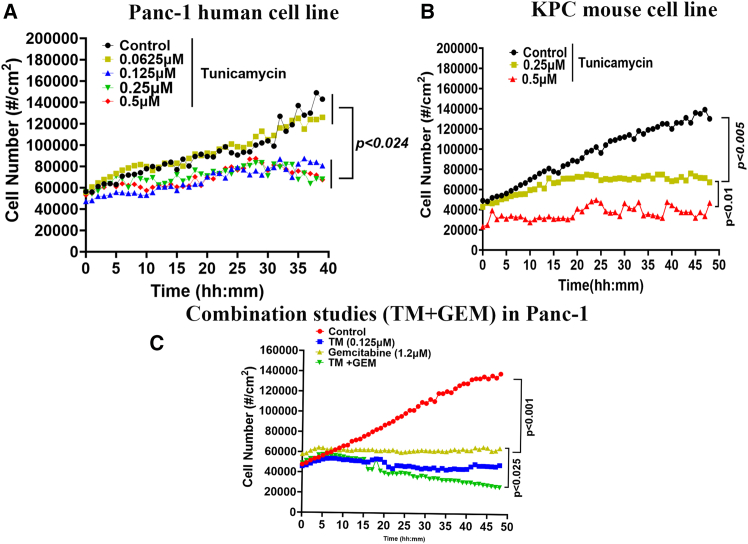
The automated single-cell proliferation assay in PDAC cell culture using phase holographic imaging system (A–C) The imaging system measured the effect of tunicamycin alone on cell proliferation of Panc-1 (A), KPC cell lines (B), and combination of gemcitabine in Panc-1 cells (C) every hour interval for 48 h in three spots of a well-following treatment. The data represent mean of three spots of a well.

The single-cell proliferation kinetic assay aimed to unravel the time- and dose-dependent effect of ^NP^TM on Panc-1 and KPC cell lines chosen for their K-RAS ^G12D^ mutations. The results demonstrated that ^NP^TM treatment effectively halted the proliferation of pancreatic cancer cells in a time-dependent manner. Notably, Panc-1 cells exhibited a lower sensitivity to ^NP^TM than KPC cells (Figures 3A and 3B). Our next step was to explore the effect of ^NP^TM in combination with gemcitabine (GEM; 0.5 μM) in GEM-resistant Panc-1 cells.^44^ We found a significant synergy of ^NP^TM and GEM following 48 h of treatment (Figure 3C).

**Figure 4:**
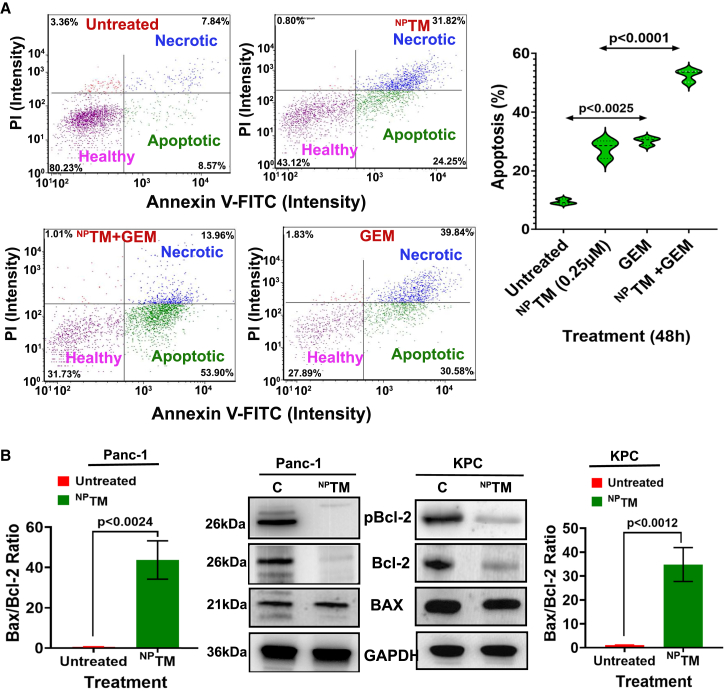
Effect of ^NP^TM, GEM, and combination on apoptosis in Panc-1 cells (A) Annexin V/PI apoptosis assay. The violin graph represents the percent apoptosis in different samples. *n* = 3/sample. (B) Estimated the levels of Bax/Bcl-2 ratio in ^NP^TM-treated and untreated PDAC cells. Data represent mean ± SD of three experiments.

TM is an apoptosis inducer in various cancer cells.^20^^,^^45^ In this study, we determined if the cell growth inhibition by ^NP^TM is due to apoptosis. The untreated and ^NP^TM-treated (0.5 μM/48 h) cells were analyzed by flow cytometry using Annexin V/PI apoptosis assay. The results showed that ^NP^TM treatment significantly elevated apoptosis in Panc-1 cells compared to the untreated group (Figure 4A). A combination of ^NP^TM and GEM treatment unveils an additive effect on Panc-1 cells.

Bcl-2 plays a crucial role in suppressing apoptosis by inhibiting the pro-apoptotic protein Bax, which is responsible for initiating the cell death program. When Bcl-2 expression decreases, there is an increase in cell death.^46^ Therefore, the ratio of Bax to Bcl-2 serves as an indicator of cell fate following drug treatment. A higher Bax/Bcl-2 ratio suggests that cells undergo apoptosis, while a lower ratio indicates that cells are alive.^47^ In our study, we investigated the effect of ^NP^TM on Bcl-2 and Bax expressions in PDAC cells (Panc-1 and KPC). We found that ^NP^TM decreases the production of phosphorylated Bcl-2 (pBcl-2) and Bcl-2 itself without affecting Bax expression in PDAC cells. As a result, the Bax/Bcl-2 ratio was higher in the treatment groups (Figure 4B). These findings suggest that ^NP^TM induces apoptosis by increasing the Bax/Bcl-2 ratio.

### TM impairs molecular heterogeneity in Panc-1 cells

**Figure 5:**
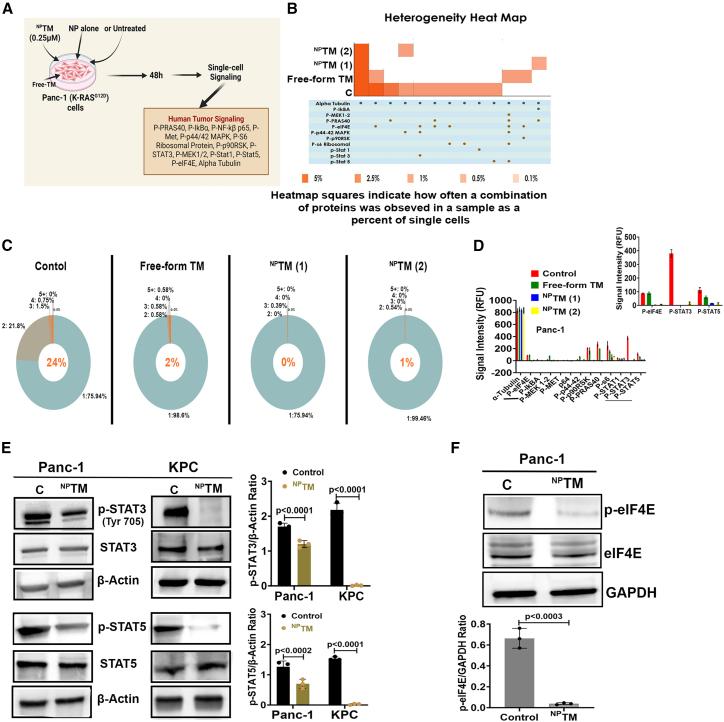
Analysis of molecular heterogeneity in Panc-1 cells following TM treatment (A) Diagrammatic presentation of experiments performed in this study. (B) Heterogeneity heatmap comparing molecular heterogeneity in untreated and treated samples. (C) Donut plot showing the percentage of polyfunctional cells. (D) Bar graph showing protein levels in (B) and (C) following treatments. (E and F) Western blotting of phospho- and regular EIF4E, STAT3, and STAT5 in PDAC cells. *n* = 3. Data represent mean ± SD.

**Figure 6:**
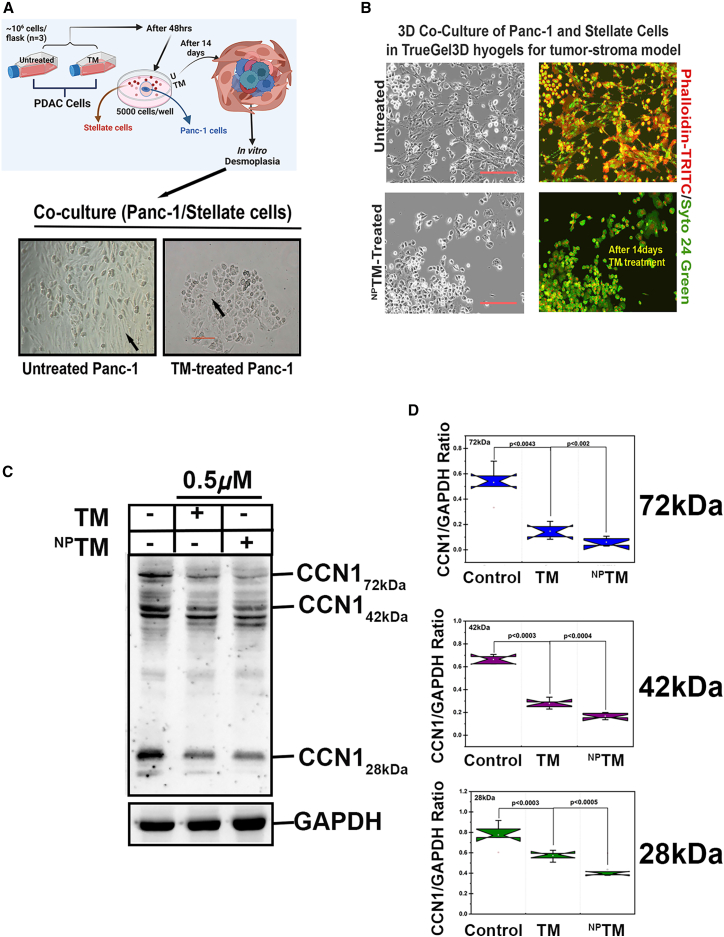
Effect of TM on 3D co-culture as *in vitro* desmoplasia and CCN1 expression in PDAC cells (A) *In vitro* desmoplasia assay using co-culture of untreated or ^NP^TM-treated Panc-1 cells and fibroblast cells as depicted in upper panel. Arrow indicates a desmoplasia-like structure. Scale bars, 50 μm. (B) 3D co-culture of Panc-1 and pancreatic Stellate cells in True Gel3D hydrogel for the detection of tumor-stroma interaction and F-actin expression. Scale bars, 50 μm. (C) Immuno-western blot analysis of CCN1 in untreated, free, and nanoencapsulated TM-treated Panc-1 cell extracts. Cells were treated for 48 h, and 50 μg total protein/sample was separated in SDS-Page gel. (D) The graphs illustrated the signaling status of CCN1 in different samples derived from five independent experiments of each group. Data represent mean ± SD. The *p* value was calculated using the student t test*.*

Molecular heterogeneity in cancer cells presents significant challenges to effective therapy because it governs adaptive response and resistance involving hypoxia, cancer stemness, and desmoplasia.^48^ In this study, we investigated whether TM regulates cancer cell heterogeneity. Using a single-cell intracellular tumor signaling analysis using Isoplexis, we found that free-TM and ^NP^TM markedly reduced molecular heterogeneity in Panc-1 cells following 48 h of treatment (Figure 5). These cells exhibit a 24% heterogenetic population (Figure 5), and among them, we observed an enrichment of phosphoproteins involved in oncogenic Kras-dependency.^49^^,^^50^^,^^51^ The heterogenicity of Panc-1 cells was diminished by TM treatment. However, the effect was more pronounced in ^NP^TM-treated cells (Figures 5C and 5D). Consistent with the data of single-cell phosphoproteomic analysis, we observed that the levels of p-STAT3, p-STAT5, and p-eIF-4E proteins were significantly reduced in ^NP^TM-treated Panc-1 cells using western blot analysis (Figure 6).

### TM blocks *in vitro* desmoplastic reactions and their prime regulator CCN1 glycoprotein expression in PDAC cells

**Figure 7:**
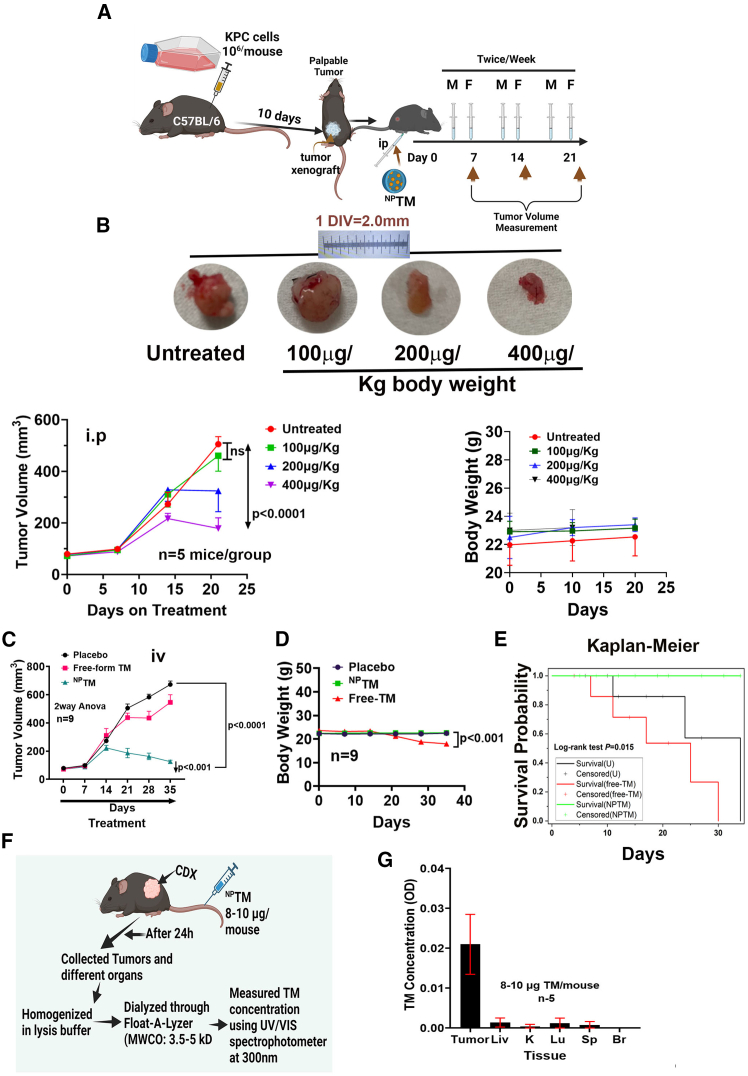
^NP^TM treatment inhibits KPC-CDX tumor growth (A) The illustration presents the treatment profile of ^NP^TM in KPC-cell implanted subcutaneous (CDX) tumors. (B) Estimated the effective tolerated dose of ^NP^TM in inhibition of tumor growth with showing no effect on body weight. Tumors were excised, and photographs were taken at the termination of the experiment. (C) Tumor growth curves of CDX tumors following i.v. injection of free form of TM and ^NP^TM or left untreated. (D) Body weight measurement. (E) Probability of survival was plotted using a Kaplan-Meier plot. (F) Experimental model for the detection of TM in tumors and different organs following i.v. injection for 24 h. (G) TM concentrations in tumors and different organs after 24 h of ^NP^TM treatment. Concentrations were measured using UV-VIS spectrophotometer.

Desmoplasia is a common characteristic of PDAC and poses a significant challenge in delivering drugs to their target.^52^ To explore whether TM could inhibit desmoplasia, we conducted an *in vitro* desmoplastic assay, as described earlier^53^ and illustrated in Figure 7A. Our results showed that co-culturing healthy PDAC cells with fibroblast cells resulted in a desmoplastic-like structure (indicated by the arrow). However, this structure was impaired when the PDAC cells were pre-treated with ^NP^TM for 48 h before co-culturing with fibroblasts (Figure 7A). This result suggests that TM suppresses the desmoplastic reaction in PDAC cells.

Cell proliferation and migration play a significant role in developing desmoplastic reactions within the tumor ecosystem.^54^ The actin cytoskeleton regulates these dynamic cellular processes, including cell division and migration, composed of filamentous actin (F-actin) and other components.^55^ Our 3D co-culture studies have shown that ^NP^TM inhibits F-actin synthesis in PDAC and stellate cells (Figure 7B). This suggests that the effects of TM on these cells may reduce the ability of F-actin to convert chemical energy into the mechanical energy necessary for cellular migration and proliferation.^55^

The key role of CCN1 glycoprotein in regulating desmoplasia in pancreatic cancer has been previously proven by us.^53^ In this study, we investigated the effect of free-TM and ^NP^TM on CCN1 protein expression in Panc cells using western blotting. CCN1 is a glycoprotein; thus, antibodies for CCN1 are always immunoreacted with a 40–45 kDa protein. However, antibodies often recognize high- (∼72 kDa) and low (∼28 kDa)-molecular-weight proteins under reducing conditions (Figure 7C). We found that free-TM and ^NP^TM treatment significantly suppressed the expression levels of these two forms of protein along with its original form (42 kDa) (Figures 7C and 7D), and ^NP^TM effectively suppressed the high-molecular-weight form of CCN1 than that of free-form TM. This observation indicates that TM significantly inhibits glycosylation in CCN1 protein, disrupting protein maturation.^56^

### The dose-dependent effect of ^NP^TM on subcutaneous tumor xenograft growth in mice

**Figure 8:**
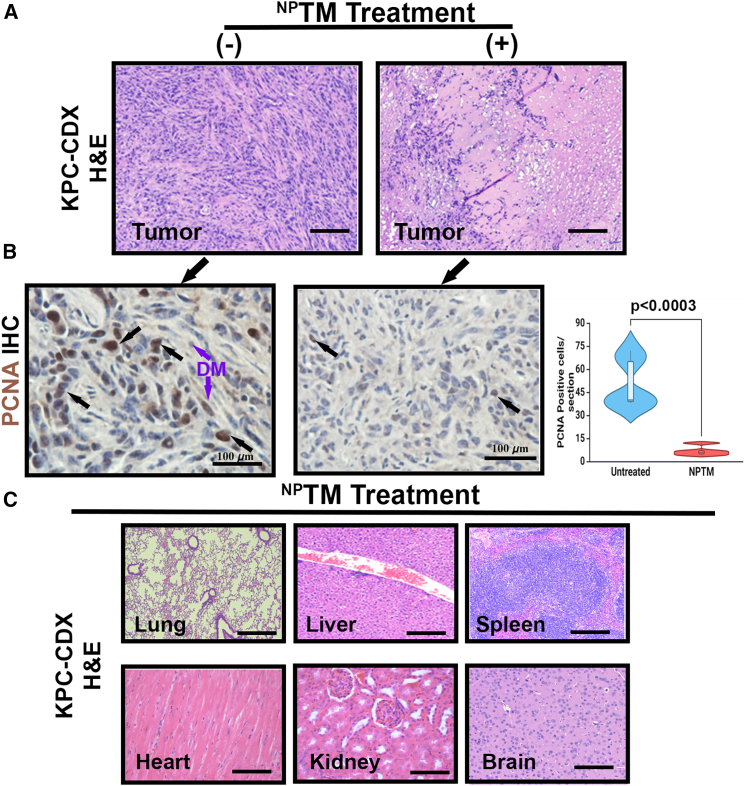
Representative of H&E and PCNA IHC with TM concentrations in tumor and different organs (A) H&E staining of untreated and ^NP^TM-treated KPC-cell-derived xenograft tumor (KPC-CDX) sections. Scale bars, 50 μm. (B) PCNA immunohistochemistry in untreated and ^NP^TM-treated KPC-CDX sections. Bar graph showing the statistical difference of PCNA in untreated and untreated samples. Data represent mean ± SD. *n* = 5. Scale bars, 50 μm. (C) H&E staining in different organs of KPC-CDX mice upon ^NP^TM treatment. 40×. Scale bars, 50 μm.

To establish the effective dose of ^NP^TM, we tested the dose-dependent effect of ^NP^TM (two times a week by intraperitoneal [i.p.] injection) in a pancreatic cancer cell (KPC) subcutaneous transplanted xenograft (CDX) model of a tumor. Weekly measurements of tumor volumes revealed that tumors in the 400 μg/kg of ^NP^TM-treated mice (*n* = 5) continued to reduce significantly throughout 25 days compared to untreated mice (Figures 8A and 8B), without affecting the body weight of the mice.

Next, we investigated the effect of free-TM and ^NP^TM on CDX tumor growth, distribution of the drugs in different organs, and toxicity. The tumor growth analysis studies using intravenous (i.v.) injection showed that ^NP^TM (400 μg/kg in 100 μL Matrigel/PBS twice a week) treatment for 35 days significantly reduced the volume of the xenograft tumors than free-TM and untreated groups (Figure 8C). Although free-form TM reduces tumor growth, due to the toxic effect of the drug, body weight was reduced significantly (Figure 8D), and three out of five5 died. At the same time, the rest of the animals were unhealthy and reaching endpoint criteria (Figure 8E). In this study, tumor growth in untreated mice reached endpoint criteria.

Furthermore, ^NP^TM therapy also notably improved survival rates compared to free TM and untreated samples. The ^NP^TM-treated mice showed no symptoms or morbidity during the experimental phase. These findings suggest that TM therapy inhibits tumor growth and enhances survival (Figure 8E).

The release studies of TM from ^NP^TM showed that TM concentration was higher in tumors than in other organs. No drug was detected in the brain (Figure 8G). The observed reduction of tumor growth by ^NP^TM (Figures 8B and 8C) was consistent with histological and PCNA IHC findings, indicating TM significantly reduced PCNA-expressing cell numbers (Figures 8A and 8B).

Finally, the toxicity analysis showed that ^NP^TMs do not trigger significant toxicity in different organs (e.g., lungs, liver, spleen, and brain) after prolonged treatment (Figure 8C). Collectively, these experiments indicate that further studies are warranted.

## Discussion

Multiple studies from different disciplines over the last several years have revealed the complexity of PDAC and led to efforts to identify new targets based on biological and genetic perspectives.^57^^,^^58^^,^^59^^,^^60^ However, acquired heterogeneous mutations and potential adverse effects in the PDAC population limit the effectiveness of these approaches. This proof-of-concept study, which explored a new treatment option by nanoencapsulating tunicamycin (TM), has yielded promising results. These positive findings not only highlight the potential of the encapsulation of an old drug TM to become more broadly efficacious in inhibiting PDAC cell survival and destroying molecular heterogeneity but also instill hope for a future with no or little side effects as survival of mice increases, which is missing in free-TM. The added benefit of targeting the tumor stroma/desmoplasia, which is genetically more stable and prevents drug delivery in PDAC, further enhances the optimism for the future of PDAC therapy.

TM is an antibiotic drug produced by *Streptomyces lysosuperificus*. It is an ER-stress inducer in a dose and time-dependent manner. A short exposure, about 6 h, promotes ER stress, while more prolonged exposure may cause ER-stress-mediated cell death.^61^ Multiple studies considered TM a potent antitumor drug in various cancers and may promote chemosensitivity.^61^ However, it was found toxic in clinical settings. We found the toxicity of the TM can be repealed if the drug is encapsulated in a biodegradable pH/hypoxic-responsive iRGD-decorated nanoparticle. Consistent with previous reports,^23^^,^^26^ TM was successfully encapsulated with pH-responsive di-block-copolymer-derived nanoparticles tagged with iRGD. The studies showed cellular uptake and distribution of nanoparticles in both normoxic and hypoxic conditions but were significantly elevated in the hypoxic environment compared to normoxic conditions. Moreover, ^NP^TM was functionally superior to free TM. It effectively blocks *in vitro* PDAC cell growth via apoptosis with lower IC_50_ of ^NP^TM, and the combination of ^NP^TM and gemcitabine treatment unveils an additive effect on PDAC cells. Induction of apoptosis represents the principal mechanism of eliminating malignant cells.^62^^,^^63^ Thus, free-TM or ^NP^TM treatment appears to be a promising strategy for PDAC therapy.

Molecular and cellular heterogeneity between cancer cells arises from complex genetic, epigenetic, and metabolic modifications, helping them to gain adaptability under stressed conditions. The heterogeneity remains a barrier for therapies, as it promotes drug resistance.^64^^,^^65^^,^^66^ In PDAC, tumor mutations/molecular heterogeneity are within a single pathway, mutant K-Ras, which plays a dominant role in the progression of the disease and constitutes a therapeutic barrier.^66^ Using phosphoproteomic *in vitro* cellular analysis, we report here that free-TM or ^NP^TM impaired the heterogeneity in PDAC cells, revealing its potential to contribute to drug sensitivity and to be combined with PDAC front-line therapies.

Desmoplasia, a universal pathobiological feature of pancreatic ductal adenocarcinoma (PDAC), is a significant barrier to therapies.^67^ CCN1 signaling, a crucial regulator of desmoplasia,^53^^,^^67^ has been a focus of our *in vitro* studies. We discovered that the desmoplastic reaction machinery, including CCN1 expression, stellate cell growth, and F-actin protein synthesis, were effectively blocked by TM. These findings reinforce the potential of ^NP^TM treatment as a promising therapeutic strategy to enhance sensitivity in PDAC through diverse molecular mechanisms. This opens an exciting avenue for further investigations, inspiring us to continue our efforts in the fight against PDAC.

Although TM is considered a potent antitumor drug, the major weakness, as we discussed in the preceding sections, is that TM is not cell-type specific, causing toxicity following TM therapy. Thus, TM research in clinical settings has declined. To overcome the limitation, the drug delivery methods have been modified in this study by using pH and hypoxic responsive nanoparticles that only released the drugs in low pH or the hypoxic zone, which is prevalent in PDAC and other aggressive cancers.^23^^,^^26^ The KPC xenograft mouse model studies confirmed the most effective dose of ^NP^TM (400 μg/kg body weight) that significantly reduced tumor growth and increased the survival probability of tumor-bearing mice following 35 days of treatment without having detectable side effects in various organs. The distribution of TM was localized in the tumors. Consistent with the previous report,^61^ we found free-TM showed toxicity with weak survival probability.

In summary, our studies have demonstrated the potential of targeted encapsulated TM to overcome the toxicity of free TM, leading to a significant reduction in PDAC xenograft tumor growth and an increase in mouse survivability. Our *in vitro* studies suggest that TM is a robust inhibitor of the heterogenic properties of PDAC cells and the desmoplastic reaction, achieved by suppressing multiple mK-Ras^G12D^-dependent signaling pathways and the CCN1-signaling pathway. These studies strongly support future mechanistic and pre-clinical research testing ^NP^TM combinations of CCN1 and K-Ras^G12D^ inhibitors and first-line chemotherapies.

### Limitations of the study

The research demonstrating the pH-responsive targetability of TM nanoencapsulation to pancreatic ductal adenocarcinoma (PDAC) cells represents a significant advancement in drug discovery for pancreatic cancer. However, the conclusions drawn from these studies are based solely on a subcutaneous tumor xenograft mouse model. To validate this hypothesis, it is essential to utilize additional models, such as organoids and orthotopic models. Furthermore, assessing the maximum tolerated dose (MTD) and pharmacokinetics (PK), both alone and in combination with other drugs, is crucial for clinical applications. Future studies that incorporate multiple models and parameters will enhance our understanding of drug-drug interactions and their molecular functions.

## Materials and methods

### Regents and antibodies

All chemicals were purchased from Sigma-Aldrich, and anhydrous solvents were obtained from VWR and Millipore. The drug information (Tunicamycin, TM) and targets were obtained from Drugbank version 5.1.12; the Drugbank accession number is DB13172 as a small molecule. TM (CAS No. 11089-65-9 and PO # 654380) was purchased from Sigma-Aldrich, USA. For *in vitro* studies, TM was dissolved in DMSO, followed by adding DMEM culture medium for the subsequent studies. TM solution in DMSO was further dissolved in sterile PBS for animal studies. Anti-rabbit IgG (# 70745) was purchased from Cell Signaling, USA; anti-mouse IgG (# sc-516102) from Santa Cruz, Inc, USA; CCN1 polyclonal antibody (#26689-1-AP) and Neuropilin-1 (NRP-1) from ProteinTech, USA; and pSTAT3 polyclonal antibody (#9145) and STAT3 polyclonal antibody, peIF4E and EIF4E antibodies, and PCNA rabbit polyclonal antibody (#13110T) from Cell Signaling, Inc., USA.

### Cell culture

The human pancreatic cancer cell lines, Panc-1, Mia-PaCa-2, BxPC-3, Capan-1, and AsPC-1, were procured from the American Type Culture Collection (ATCC). Panc-1^CCN1KO^ stable cell line and mouse pancreatic cancer cell line, KPC, were generated and maintained in our laboratory. Panc-1, Panc-1CCN1KO, and KPC cell lines were cultured in high-glucose DMEM medium (Thermo Fisher Scientific) with 10% fetal bovine serum (FBS) and 1% v/v Penicillin-Streptomycin (pen-strep) at 370°C in a humidified incubator containing 5% CO2. The other cells are maintained in regular DMEM with 10% FBS and Penicillin-Streptomycin. The cell lines were subcultured using enzymatic digestion of 0.25% trypsin/1mM (Thermo Fisher Scientific) upon reaching 70% confluency.

### Generation of pH and hypoxic response iRGD-tagged TM-nanoparticle

pH/Hypoxic responsive iRGD-conjugated TM nanoparticles were designed based on our recent published methods^23^^,^^26^ with brief modification of earlier work.^68^^,^^69^ Briefly, bis (methoxy propionic acid) or bis (MPA) was used to generate polymer precursor for synthesizing PEG-b-poly (carbonates) via a ring-opening polymerization reaction. The PEG-b-poly(carbonates) were modified with 2-pyrrolidine-1-yl-ethylamine (pK_a_ = 5.4) to design a pH-responsive block polymer, PEG-PY referring to PEG-b-poly(carbonates) copolymer with the poly (carbonate) segment appended with 2-pyrrolidin-1-yl-ethyl-amine side chains. The synthesized polymer was primarily characterized by Fourier transform infrared (FTIR) spectrometry and 1H nuclear magnetic resonance (NMR) spectroscopy.

After generating the hypoxic-responsive block copolymer PEG-py, TM, a hydrophobic drug,^70^ was encapsulated within the nanoparticles composed of this copolymer according to previous work.^23^ For this, the copolymer and the drug were dissolved in DMSO and dropwise added to PBS buffer with pH 7.4 followed by dialyzing using a Float-a-Lyzer and filtering through a PES filter (0.2 μm) to collect the nanoparticle-encapsulated TM as the filtrate. The nanoparticle suspension was used to measure the particle size and surface charge (or zeta potential) using dynamic light scattering (DLS). In addition, Alexa Fluor 647 was also co-encapsulated to study the cell trafficking of nanoparticles.

To make targeted pH/hypoxic responsive nanocarriers, the iRGD (CRGDKGPDC) peptide (C_35_H_57_N_13_O_14_S_2_) was conjugated to PEG-py using “click” chemistry as described earlier.^26^

Finally, the release of the TM from PEG-py was detected by dialyzing the product with PBS at different time points under normoxic and hypoxic conditions using a float-a-layer dialysis system (MWCO 3.5–5 kDa).

### Drug release study of ^NP^TM in cell-free system

The TM released from the targeted nanoparticles was measured using a dialysis-based experiment employing Float-a-Lyzer type device (MWCO 3.5–5 kDa) as described previously by us24. Briefly, 1 mL of ^NP^TM solution was placed inside the float-a-layer, and the suspension was dialyzed against lysis buffer under hypoxic, normoxic, or low pH conditions (pH 6.2) using controlled CO2 incubator. The amount of TM released from the carrier system was measured in the bulk phase by withdrawing a specified volume (1 mL) of the solution from this phase periodically and replacing it with an equal amount of fresh media to maintain the sink condition as described.^23^^,^^26^ The drug concentration was measured at 300 nm using a plate reader after determining the λmax via a UV/VIS spectrophotometer scanning from 250 to 350 nm (Figure S1).

### *In vitro* stability in plasma

The *in vitro* stability of the TM was determined in healthy mouse plasma (Fisher Scientific, CAT # 50-203-5347) as per previous method.^71^ Briefly, mouse plasma was diluted to 80% with 0.05 M PBS (pH 7.4) at 37^o^C. Free-TM or ^NP^TM with final concentration of 1 μM/mL was added in pre-heated healthy mouse plasma at 37°C for varying lengths of time ranging from 0 to 19 h. The assays were performed in a CO_2_ incubator at 37°C and conducted in triplicate. Following incubation, 50 μL samples were collected and the plasma deproteinized by adding 200 μL acetonitrile. The samples were vortexed for 1 min and centrifuged at 4°C for 15 min at 14,000 rpm. The clear supernatants were analyzed for the TM concentration using UV-VIS spectrophotometer at 300 nm (Figures S1 and S2). The values represent the mean of three independent experiments. The *in vitro* plasma half-life (t1/2) of TM was calculated using the expression t_1/2_ = 0.693/b, where b is the slope found in the linear fit of the natural logarithm of the fraction remaining of the parent compound vs. incubation time.^71^

### *In vivo* TM-release studies

To measure the TM concentrations in xenograft tumors and different organs of the mice, ^NP^TM containing 8–10 μg of TM was injected intravenously into the mouse (*n* = 5) for 24 h. Tissues were collected, lysates were prepared using RIPA buffer (Cell Signaling Technology), and protein concentration was measured using BSA quantification kits. Tissue lysates were placed inside the float-a-layer and dialyzed against water for 2 h. In dialyzed samples, TM concentrations were measured using a UV-VIS spectrophotometer at 300 nm (Figure S1).

### *In vitro* desmoplasia

To investigate the effect of TM on the desmoplastic reaction, we utilized a well-established *in vitro* desmoplasia model with some modifications.^53^ In brief, we seeded 5,000 TM-treated and untreated Panc-1 cells in the central well of a central well culture dish (Corning, CAT# CLS3260) and incubated them at 37°C in a CO2 incubator with high-glucose DMEM supplemented with 10% FBS for 24 to 48 h. Once the Panc-1 cells reached 60%–70% confluence, we added 30,000 human pancreatic stellate cells (HPaSteCs) in the outer part of the central ring of each Petri dish. Fibroblast culture media were then added to cover both cell cultures by replacing the DMEM. The central ring serves as a barrier to prevent the mixing of tumor cells and HPaSteCs during the seeding process. The culture was maintained for 4 to 7 days, after which the cultures were fixed in methanol. Observations were made, and photographs were taken using a Nikon microscope.

### Co-culture of Panc-1 and pancreatic stellate cells in True Gel3D hydrogel

The True Gel3D hydrogel technique was performed according to the manufacturer’s instructions (Millipore Sigma). In brief, water, TrueGel3D, FAST-DEXTRAN, and the TrueGel3D RGD integrin adhesion peptides were mixed thoroughly. The mixture was incubated for 5 min to allow the RGD peptide to attach to the maleimide groups of the FAST-DEXTRAN polymer. Next, untreated and ^NP^TM-treated (0.5 μM) Panc-1 cells and HPaSteCs were added to the True Gel3D mixture. A total of 27 μL of the mixture was then placed into a culture, allowing approximately 3 min for the gel to form. Following this, fibroblast culture medium was added in sufficient volume to cover the gel. The dish was incubated for 1 hour in a CO_2_ incubator, after which the medium was replaced with fresh cultured media, which was changed frequently over the course of 14 days as needed. The cells were fixed using 4% paraformaldehyde in PBS (with Ca^2+^/Mg++) for 1 h and washed four times for 5 min each in PBS (w/o Ca^2+^/Mg++). The hydrogels were subsequently incubated with 0.5% (v/v) Triton X-100 in PBS for 10 min and washed three times (10 min each) in PBS. For F-actin staining, hydrogels were incubated with 1.7 μg/mL phalloidin-TRITC in PBS for 1.5 h in the dark and washed three times (5 min each) in PBS. For nuclei staining, gels were stained with 17 μmol/L Syto24 Green (Invitrogen) for 30 min at room temperature in the dark. Finally, images were captured using epifluorescence microscopy. In the images, red represents the actin cytoskeleton, and green represents the nuclei.

### Western blot analysis

The western blot analysis was the same as described earlier.^16^^,^^26^^,^^72^ Briefly, the cell lysates were prepared using RIPA buffer, and protein concentration was measured using BSA quantification kits. Thirty to fifty microgram protein of each sample was separated in SDS-PAGE gel electrophoresis followed by semi-dry transfer into nitrocellulose membrane using a Trans-Blot Turbo Transfer System (Bio-Rad). Finally, membranes were probed with antibodies treated with peroxidase-conjugated secondary antibody and then visualized by enhanced chemiluminescence.

### Cell viability assay for the detection of IC_50_ of free-TM and ^NP^TM and monitoring effects of drug combinations

The therapeutic effect of free- and encapsulated TM and GEM in human (Panc-1) and mouse (KPC) cell lines was measured in a checkerboard fashion. Cells (1 × 10^4^) were seeded into a 96-well plates with eight wells/treatment and incubated overnight. Next day, cells were treated with different doses of free-TM, ^NP^TM, and a single dose combination of TM and GEM. Untreated control cells were treated with DMSO. Cell viability was determined after 48 h using crystal violet cell viability assay. Results were graphed, and IC_50_ was measured using GraphPad prism 10 software, according to the formula: Y = 100/1 + 10ˆ((LogIC50-X)∗Hillslope) where X = log of dose, Y = growth inhibition value normalized to control, and Hillslope = unitless slope factor or Hill slope.

The automated single-cell proliferation assay by Phase Holographic Imaging System was used for testing the dose-dependent effect of TM and drug combination impact on pancreatic cancer cells in real time. Representative data from biological replicates were shown in this study.

### Immunohistochemistry

Immunohistochemistry (IHC) was carried out using our previous method.^73^ Briefly, tissue sections (5-μm thick) were deparaffinized in xylene, rehydrated in different grades of alcohol, and washed with PBS. The sections were incubated in heat-induced 1× antigen retrieval solution by autoclaving for 30 s followed by 30 min to bring the sections to room temperature. The sections were then blocked with a tissue blocker (Life Technologies) for 30 min at room temperature followed by incubation with polyclonal NRP-1 antibody (1:1,000) or PCNA antibody (1:1,000) overnight at 4^o^C. The sections were incubated with ImmPRESS HRP Universal secondary Antibody (Vector Laboratories, MP-7500) for 30 min and stained with DAB using ImmPACT DAB EqV substrate kit following counterstaining with hematoxylin (blue). The images were captured under a Leica photomicroscope. The PCNA positive cells were estimated from three IHC sections, and in each section, three hots spots were considered for counting PCNA-positive cells.

### Confocal microscopy

Panc-1 cells were seeded at 5,000 cells/chamber on 4-well chamber slides and allowed them to grow for 70% confluency. The cells were exposed to Alexa 674 (AF674) dye-conjugated iRGD-tagged nanoparticles for 6 h under hypoxic and normoxic conditions. Cells were trypsinized and washed with 1xPBS (pH 7.4). The percentage of labeled nanoparticle uptake by the cancer cells was analyzed using a BD Accuri C6 Flow Biosciences Cytometer. All experiments were conducted in eight sets of experiments. For hypoxia treatment, cells were placed in a hypoxic chamber containing 1% O_2_ and 5% CO_2_ balanced with N_2_ and then incubated at 37^o^C.

### Detection of apoptosis by Annexin V-FITC/PI staining kit using flow cytometry

An Annexin V-FICT/PI apoptosis detection kit (Thermo Fisher Scientific, #88-8005-74) was used to detect the apoptotic death of pancreatic cancer cells following TM treatment with the previous method.^74^ Briefly, Panc-1 cells after ^NP^TM or combination of TM and GEM treatment were dissociated as single cell by cell dissociation solution (Sigma Chemical) and collected by centrifugation at 300 g for 5 min at 4^o^C. Cells were stained with 5-μL Annexin V and 5-μL PI solution for 15 min at room temperature under dark conditions. The stained samples were analyzed by flow cytometry.

### Single-cell phosphoproteomic analysis for *in vitro* cellular heterogeneity

Panc-1 cells were treated with TM, ^NP^TM, or left untreated for 48 h; 30,000 viable cells were loaded onto the 32-plex human polyfunctional (cancer) strength single-cell IsoCode chips (IsoPlexis) and inserted into the slots of the Isolight machine for scanning reaction. The data were analyzed using IsoSpeak software (Isoplexis). The deviation in expression of some phospho-protein profiles were further verified by western blotting.

### Xenograft tumor studies

All animal studies were reviewed and approved by the Kansas City VAMC’s Animal Care and Use Committee, and all animal care was in accordance with the NIH and Institutional guidance.

The C57BL/6 mice were bred and maintained in VAMC animal facilities. To generate subcutaneous tumor, we injected 2 × 10^6^ KPC cells suspended in 100 μL media with Matrigel in 1:1 ratio into the flanks of 8-week-old male and female C57BL/6 mice (*n* = 6 or more). Once the tumors were palpable (around 50 mm^3^), the mice were randomly distributed for untreated or treated groups. The tumor volumes were recorded every 5 days, and mice were sacrificed after 4 weeks or reached to the endpoint criteria. At the end, tumors and other organs were collected for histological and biochemical analysis.

### Survival analysis

Kaplan-Meier survival curves were generated using Origin 2025 to estimate overall survival difference among untreated, free-TM, and ^NP^TM, and *p* values were calculated using the long rank test.

### Statistical analysis

All *in vitro* experiments were performed in triplicate or more, and the data are presented as the mean ± SD. Two-tailed Student t test and one-way ANOVA were used to compare continuous variables between two or more groups. In the survival data analysis, statistical comparisons were performed using Kaplan-Meier method and log rank test. All statistical analyses were performed using GraphPad software 10 and Origin 2025. *p* < 0.05 was considered statistically significant.

## Data availability

The authors affirm that all the supporting data of these studies are available within the articles and Supporting Information or from the corresponding authors on a reasonable request.

## Acknowledgments

This work was supported by VA Merit grant, USA (S.B. and S.K.B.) and by NIH/NCI P30CA168524, USA (PI: Roy Jensen, MD) (S.H.B.). We appreciate all the participants in this study and Kansas City VA Research office, Kansas City, USA and MVBRF, Kansas City, USA.

## Author contributions

Conceptualization, D.D., M.Q., S.K.B., and S.B.; methodology, D.D., M.D., S.U., A.D., A.D., A.H.B., and I.H.; formal analysis, D.D., S.B., S.K.B., and M.Q.; investigation, D.D., S.K.B., S.B., S.U., I.H., and A.B.; statistics, F.D.; resources, S.B., M.Q., and S.K.B.; writing—original draft, D.D.; writing, review, and editing, S.B., S.K.B., S.K., and S.H.B.; supervision, S.B. and S.K.B.; funding acquisition, S.B. and S.K.B.

## Declaration of interests

The authors declare that they have no competing interests. Three authors are also affiliated with University of Kansas Medical center. These authors are Sushanta K Banerjee, Snigdha Banerjee, and Inamul Haque. Part of the works have submitted as VA patent (Submission OA2024-05237).

## Supplemental information

Document S1. Figures S1–S5

Document S2. Article plus supplemental information
