# Supplementary material for: Targeted nanoencapsulation of tunicamycin reduces toxicity while improving its therapeutic effectiveness in pancreatic cancer cells
Source: Mol Ther Oncol. 2025 Sep 3;33(4):201047. doi: 10.1016/j.omton.2025.201047 (PMC12481896; doi:10.1016/j.omton.2025.201047)
Supplement: Document S1. Figures S1–S5 [file mmc1.pdf]

OMTON, Volume *33*

Supplemental information

Targeted nanoencapsulation of tunicamycin reduces toxicity while improving its therapeutic effectiveness in pancreatic cancer cells

Debasmita Dutta, Sunil P. Upadhyay, Archana De, Inamul Haque, Axel H. Breier, Alok De, Daniel J. Mettman, Suman Kambhampati, Mohiuddin Quadir, Francisco Diaz, Sushanta K. Banerjee, Stefan H. Bossmann, and Snigdha Banerjee

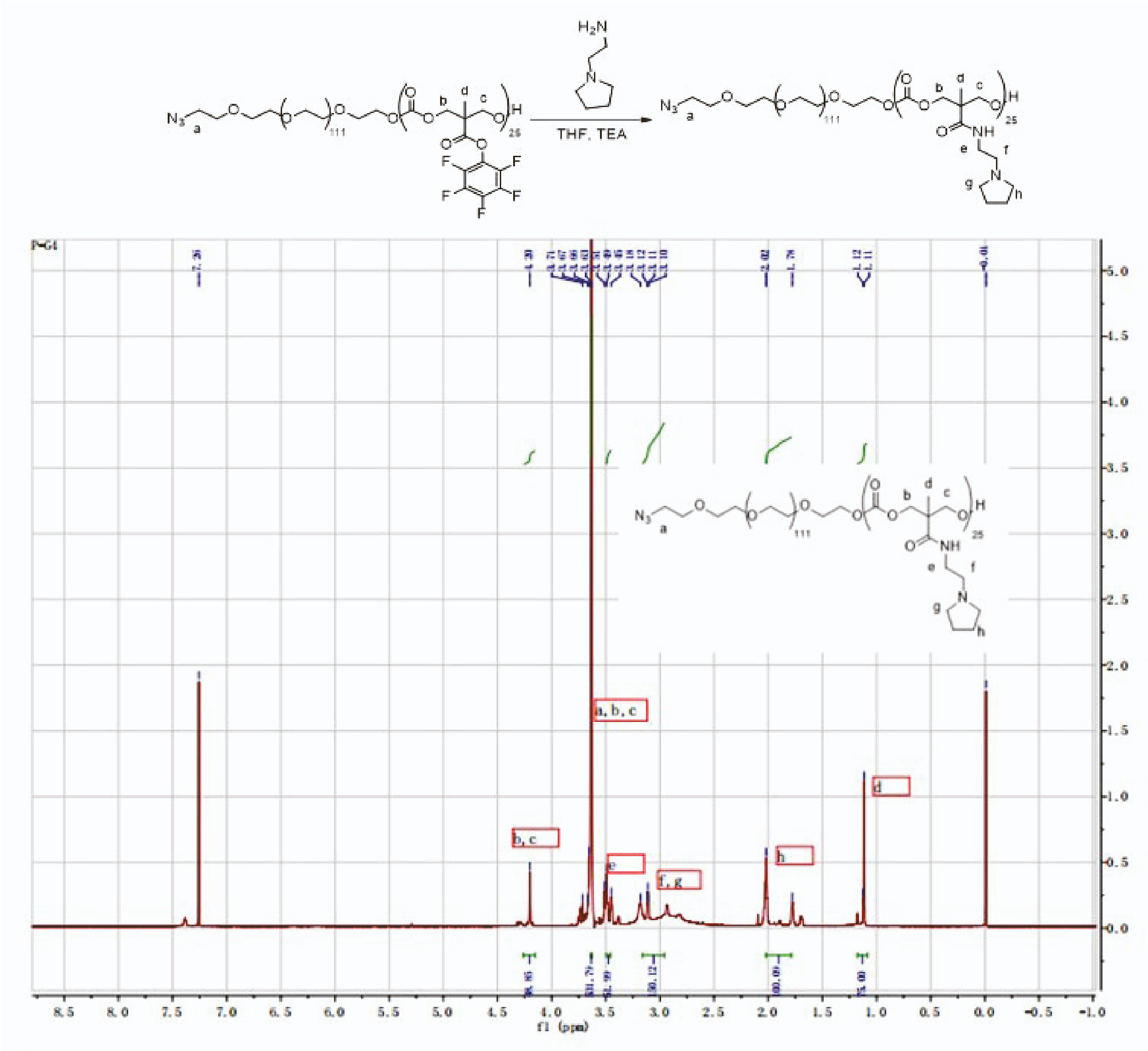

Figure S1. Characterization of an azide-based di-block polymer using NMR.

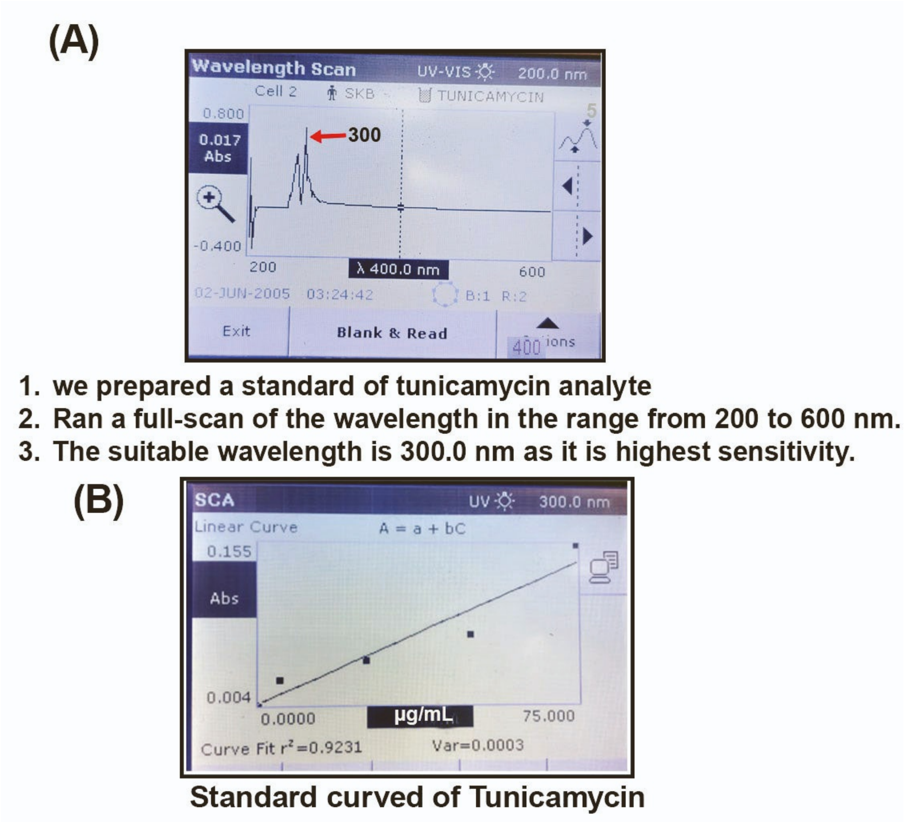

Figure S2. Detection of Tunicamycin in cell-free system. (A). detection of highest sensitive wavelength of Tunicamycin using UV/VIS spectrophotometer, and **(B).** Standard curve of Tunicamycin

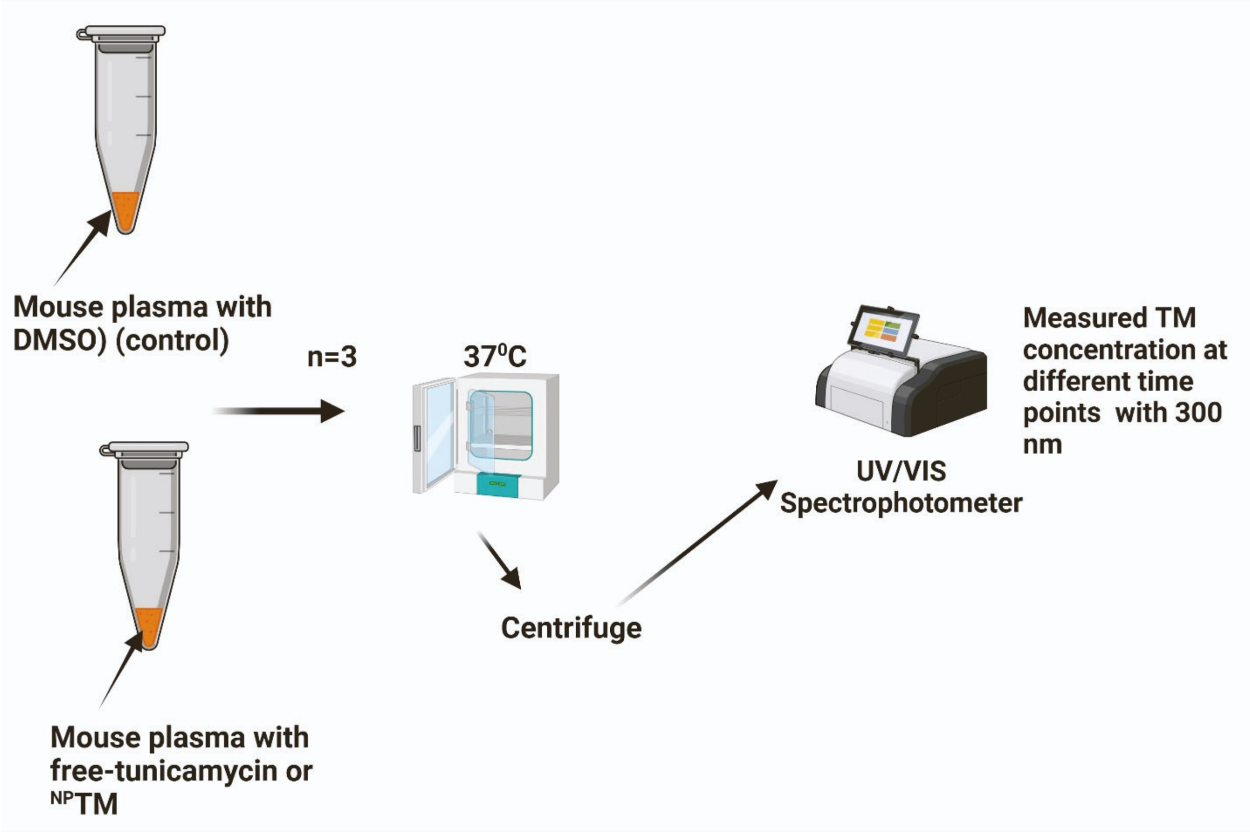

Figure S3. ***In vitro* plasma assay.** The bioavailability of Tunicamycin was measured using *in vitro* plasma assay. Tunicamycin or ^{NP}TM (1µM) in 1 mL mouse plasma (diluted to 80% with 0.05M PBS, pH 7.4) was incubated at 37ºC for different time points. After centrifugation, TM concentration was measured in a UV/VIS spectrophotometer at 300nm.

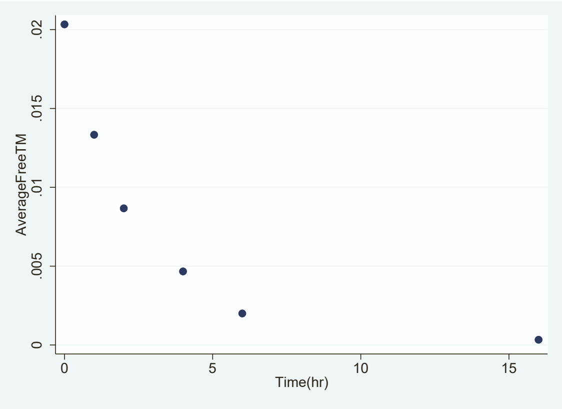

Figure S4. Half-life calculation. The in vitro plasma half-life ($t_{1/2}$) of free-TM was calculated using the expression t1/2=0.693/b, where b is the slope found in the linear fit of the natural logarithm of the fraction remaining of the parent compound vs. incubation time. We calculated

the half-life after excluding the observations at 16 hr. The obtained half-life was $t_{1/2}$ = 1.836 hr. [95% confidence interval, (1.717, 1.955)].

(A). NRP-1 Western blotting

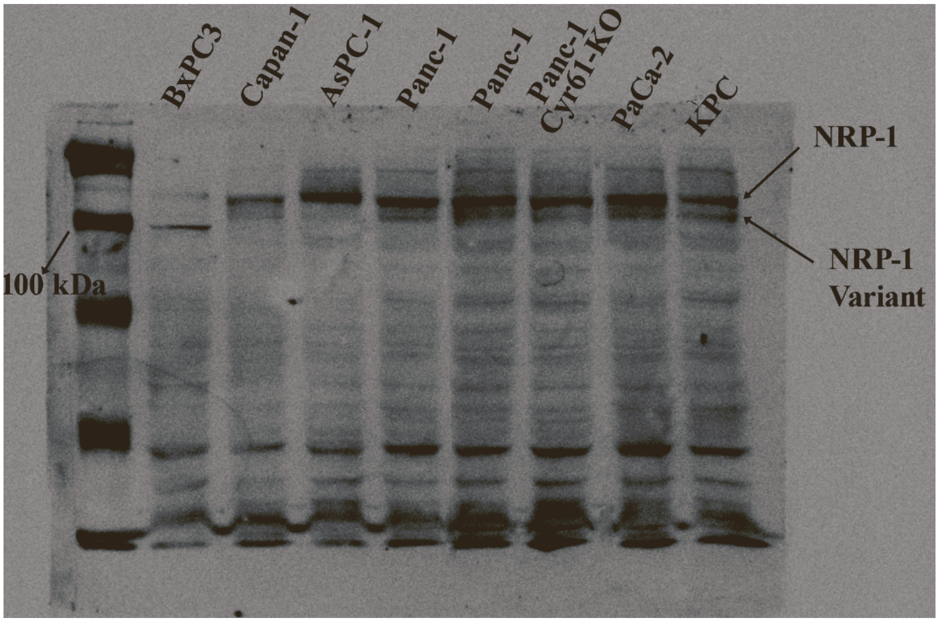

(B). β-Actin Western blotting

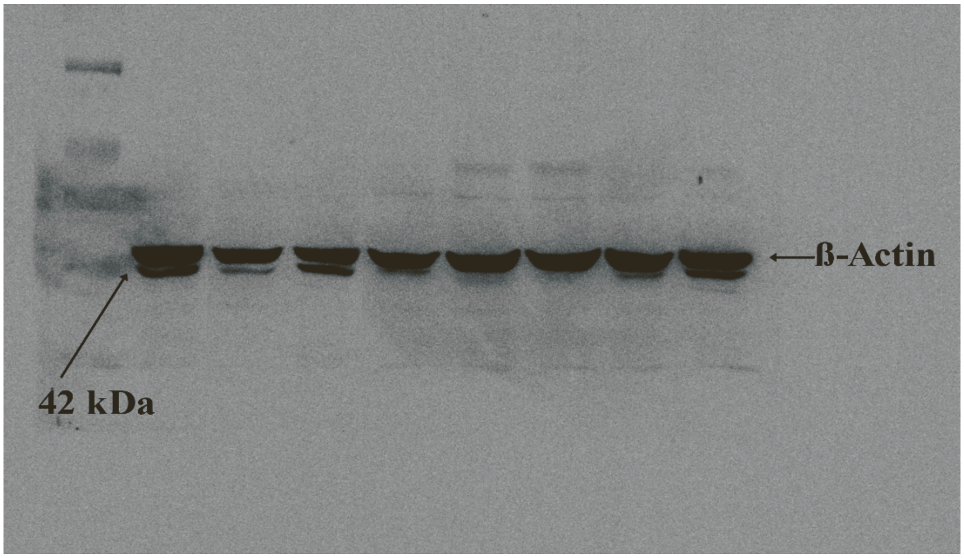

Figure S5. NRP-1and β-Actin expressions in different PDAC cell lines
